# 

**Published:** 1887-03

**Authors:** 


### CONTENTS, MARCH. 1887, VOL. II, NO. IX.

ORIGINAL COMMUNICATIONS—Congenital Stricture of Urethra; Successful Operation. By W. G. Jameson, M. D. 355
A Difficult and Complicated Obstetrical Case: By R. H. Eanes, M. D., Rice's Crossing, Texas................ 357
A Case of Hysteria in a Young Lady. By W. M. Powell, M. D., Albany, Texas.............................. 360
Abortion following Removal of a Cervical Polypus. By W. H. Lancaster, M. D., Coleman, Texas............. 363
CULLINGS FROM CONTEMPORARIES—Oxygen in Therapeutics 368
Medical History and Biography........................ 320
Therapeutic Properties of Antipyrine.................. 371
SOCIETY NOTES—Arrangements for the Coming Meeting Texas State Medical Assc........................... 374
American Medical Association......................... 374
Report of Johnson Co. Board of Censors................ 375
Proceedings of Van Zandt Co. Med. Ass'n............. 377
PERSONAL—Dr. M. M. Meyers......................... 378
BIOGRAPHICAL—The New State Health Officer, Dr. R. Rutherford, with portrait.......................... 379
NECROLOGICAL—Dr. James H. Haley.................. 380
Rev. J. Davis Gray................................ 381
EDITORIAL—The Medical Department of the University of Texas............................................ 383
The Relations of Physicians to their Medical Supplies.. 386
Banquet or no Banquet............................... 388
The Medical College Question......................... 389
Vital Statistics...................................... 390
A Disclaimer....................................... 392
MEDICAL NEWS AND MISCELLANY—A General Invitation .. 393
Texas Surgery...................................... 394
The Texas Dental Bill................................ 375
Texas Carries off the Honors......................... 395
Substitutions in Prescriptions........................ 396
Transactions Tex. State Med. Ass'n.................. 397
BIBLIOGRAPHY........................................ 398
ADVERTISERS NOTICES............................... 398

